# Evaluation of barium tungstate nanocrystals for the sorption of radioactive cobalt and europium from aqueous solutions

**DOI:** 10.1038/s41598-023-48510-w

**Published:** 2023-12-07

**Authors:** M. I. A. Abdel Maksoud, M. A. Youssef, H. S. Hassan

**Affiliations:** 1 Radiation Physics Department, National Center for Radiation Research and Technology (NCRRT), Egyptian Atomic Energy Authority (EAEA), Cairo, Egypt; 2 Hot Lab. Center, Egyptian Atomic Energy Authority (EAEA), P.O. 13759, Inshas, Egypt

**Keywords:** Environmental sciences, Chemistry, Materials science

## Abstract

Herein, barium tungstate BaWO_4_ nanocrystals were chemically prepared and then estimated as inorganic sorbent material to eliminate the radioactive cobalt and europium from the waste stream. The characterization of BaWO_4_ nanocrystals is completed over several analytical techniques. TEM and SEM images show the formation of sphere-shaped BaWO_4_ structures in the nanoscale range. Also, XRD and FTIR revealed the successful preparation of BaWO_4_. Optimum factors affected by the sorption process are determined using batch mode. Sorption equilibrium was achieved after 60 min with the initial concentration of metal ion at 100 mg/L and at optimum pH five for both radionuclides, respectively. The different kinetic models are applied. The obtained data shows that the sorption process followed a pseudo-second order. The sorption capacity for ^60^Co at pH of 5 and 25 °C is 310.6 mg g^−1^, and ^152+154^Eu is 409.9 mg g^−1^. The thermodynamic studies illustrated that the sorption process was spontaneous and endothermic.

## Introduction

During the past few decades, nuclear applications have been extensively utilized in many countries to produce electricity and various activities, producing considerable radioactive waste. So, removing the radioactive wastes has become an essential challenge that must be solved precisely^1–6^. Many radioisotopes (^128^Pd, ^137^Cs, ^235^U, ^89^Sr, ^59^Fe, ^131^I, ^57^Co, ^65^Zn, ^241^Am, etc.) are the primary contaminants present in radioactive waste generated from medicine, nuclear power plants, industrial effluents, agriculture, and research^5,7–10^. Various methods were applied to remove the radioscopies from the waste stream, such as membrane separation^5^, solvent extraction, photocatalysis^7^, ion exchange^8^, and adsorption^9,10^. The adsorption technique is vastly prestigious as a potential way to eliminate radionuclides in respect of many merits such as elevated efficiency and depressed cost as well as simple operation^9,11–15^. A broad domain of sorbent materials, such as nano metal oxides chitosan, zeolites, functionalized graphene, metal–organic frameworks (MOFs), MXene, composite materials, and Covalent organic frameworks functionalized electrodes, are available for the adsorption method^16–20^. The nanoparticles have acquired appreciable attentiveness in many applications because of their distinctive properties, such as high surface area and well-controlled facets.

The heaviest component of the alkaline earth tungstate family is barium tungstate (BaWO_4_). BaWO_4_ (BWO) crystallizes in the tetragonal scheelite-type structure at room temperature, similar to several ABX_4_-type compounds. As a result of its exceptional electrical conductivity, magnetic characteristics, and luminescence, BWO has drawn considerable interest^21^. BWO-based materials are of crucial importance in a broad range of technical applications, including light-emitting diodes^22^, humidity sensors^23^, optical filters^24^, optoelectronic^25^, dielectric materials^26^, and luminescence^27^. Recently, BWO-based materials have been used as efficient materials to remove dyes and hazardous contaminants for water treatment applications^28–31^.

To the best of our knowledge, there are no reports in the literature regarding the use of BWO nanostructured material in the sorption of radioactive cobalt and europium from aqueous solutions. In this study, BWO nanostructures were obtained by facile soft chemical technique, following their characterization to investigate their structural, microstructural, elemental, and surface characteristics by XRD, SEM, TEM, EDX, and FTIR spectroscopy. The second section looked at the capabilities of BaWO_4_ in the sorption of radioactive cobalt and europium from aqueous solutions. This research paper discusses and presents the obtained findings.

## Experimental

### Chemicals and reagents

All reagents consumed in the experiments are AR-grade chemicals and used without purification. Cobalt chloride and europium oxide have been purchased from Sigma–Aldrich Company. Solutions of all used reagents have been prepared by dissolving cobalt and europium salts in distilled water. Also, barium chloride dihydrate (BaCl_2_·2H_2_O, 99%) and sodium tungstate dihydrate (Na_2_WO_4_·2H_2_O, 99%) were used to synthesize BWO.

### Synthesis of BaWO_4_

**Figure 1:**
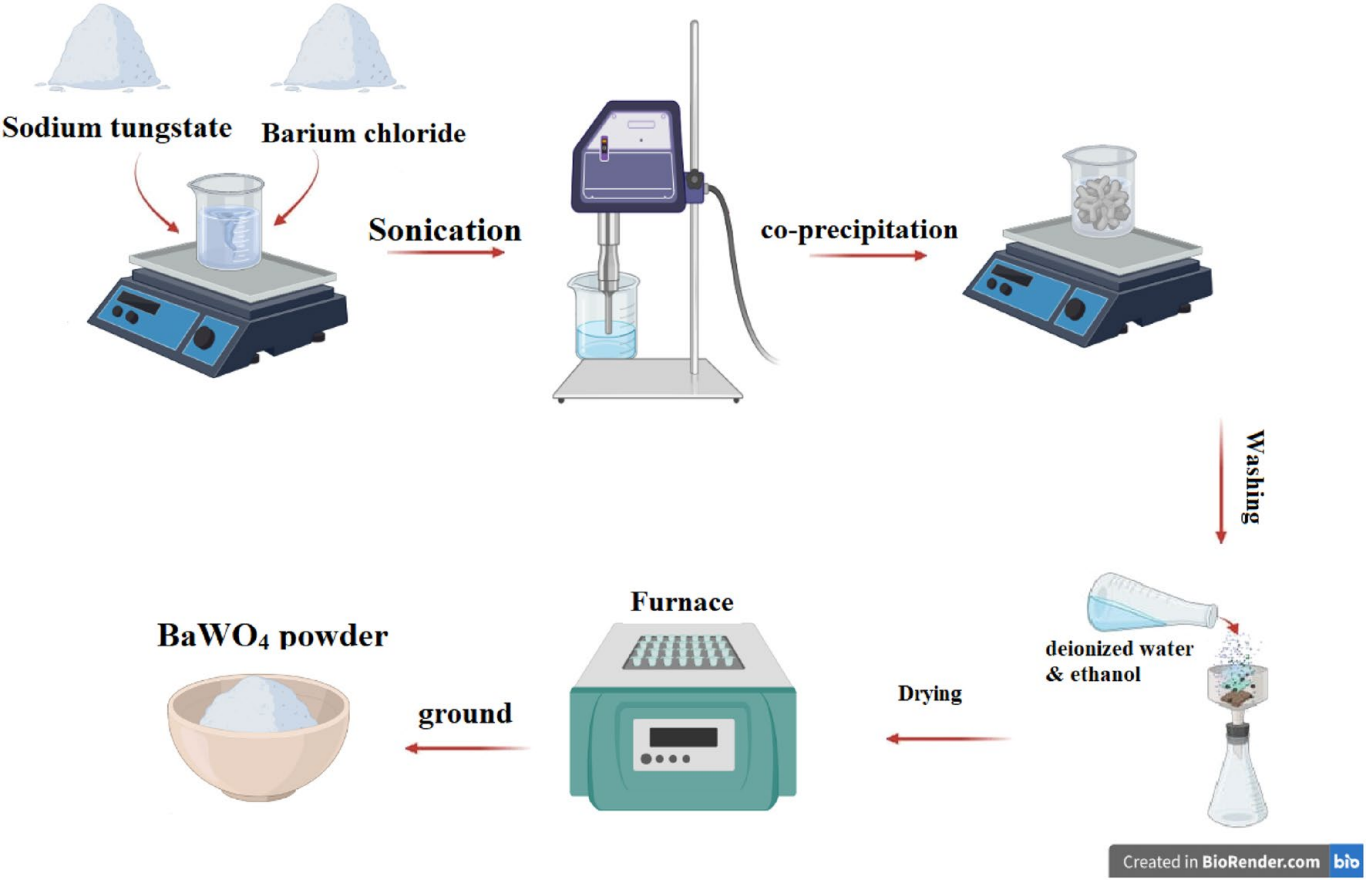
The schematic diagram for the synthesis process of BaWO_4_.

The co-precipitation process was used to synthesize barium tungstate BWO. In a typical synthesis, one mmol of sodium tungstate dihydrate and barium chloride dihydrate slats have been separately dissolved in 30 mL of distilled water in 2 different beakers. After that, the barium solution was gradually added to the other solution. Moreover, after 30 min of sonication, a white precipitate was produced and washed numerous times with ethanol and deionized water. Consequently, the product BWO was dried in a furnace for 2 h at 200 °C. Finally, the BWO was ground to obtain fine powder, as presented in Fig. 1.

### Sorption studies

The batch technique has been applied to estimate the kinetic and isotherm studies for the sorption process. To define values of pH range that maximum sorption of radioactive cobalt and europium onto the surface of BWO takes place, a group of 25 mL glass, each one comprising 0.01 g from nanomaterial and 10 mL cobalt and europium solutions, 100 mg/L. The initial pH values were modified to the extent of 2.0–9.0. This is obtained using 0.01M HCl and NaOH. The glass container was shaken at about 24h/25 °C until achieving the equilibrium case. Exploratory experiments presented that the equilibrium case for the sorption process to radioactive cobalt and europium was achieved after one hour. The solution of ions and prepared BWO were centrifuged until the loaded BWO was separated from the liquid phase; after this, one ml from the liquid phases was withdrawn. The activity of radioactive cobalt and europium has been determined by gamma-ray spectrometry by high resolution (7.5%) NaI(Tl) scintillation detector model 802–3X3, Canberra, USA

1 $$\% R = \frac{{(A_{0} - A_{t} ) \times 100}}{{A_{0} }}$$

#### Determination of point of zero charge

The batch technique was used to estimate the pH value at which the material surface's net charge is zero (the point of zero charge). A series of glass bottles containing 0.1 g of BWO powder and 10 mL of 0.1 mol/L inert electrolyte, such as KNO_3,_ was shaken for three h at different initial pH values (pH_initial_) and room temperature. After centrifugation of the two phases, the final pH values, pHfinal, of KNO_3_ solutions were determined.

#### Kinetic experiments

Kinetic experiments have been implemented at 298 K using initial concentrations, 100 mg/L, for cobalt and europium radioactive. Also, the sorption experiments have been executed at several temperatures (298, 338, and 358 K). For kinetic investigations desirable weight, 0.01 g of barium tungstate was mixed with 100 mL of cobalt and europium; the solutions were spiked with ^60^Co and ^152+154^Eu radioisotopes, respectively; these solutions were kept under stirred in the shaker, and the temperature was adjusted to a desirable value. Then the solutions were centrifuged till the separation of two phases took place, then a fixed volume of about (1 mL) of the solution was withdrawn to determine the quantity of unabsorbed radioactive cobalt and europium that exist in the solution as shown in the following equation:

2 $$q_{t} = \;\;\frac{{A_{o} - A_{t} \;}}{{A_{o} }} \times C_{o} \times \frac{V}{m}$$

q_t_ refers to the amount of both radionuclides (mg/g) at any time t, A_o_ as well as A_t_ refers to the activity for radionuclide at initial as well as equilibrium, C_o_ is the initial concentration of each radionuclide, V point out volume (L) finally m is weight in a gram of barium tungstate.

#### Equilibrium experiments

In isotherm experiments, about 10 mL from the radionuclides with varying concentrations, 50–400 mg L^−1^, has been added to 0.01 g from barium tungstate at various temperatures and an optimum pH of 5.0. After the accepted contact time (one hour) was attained, 1 mL from both radionuclides was withdrawn, and then the quantity of radionuclide held onto in barium tungstate (mg/g) was calculated.

## Results and discussion

### Characterization of barium tungstate nanospheres

**Figure 2:**
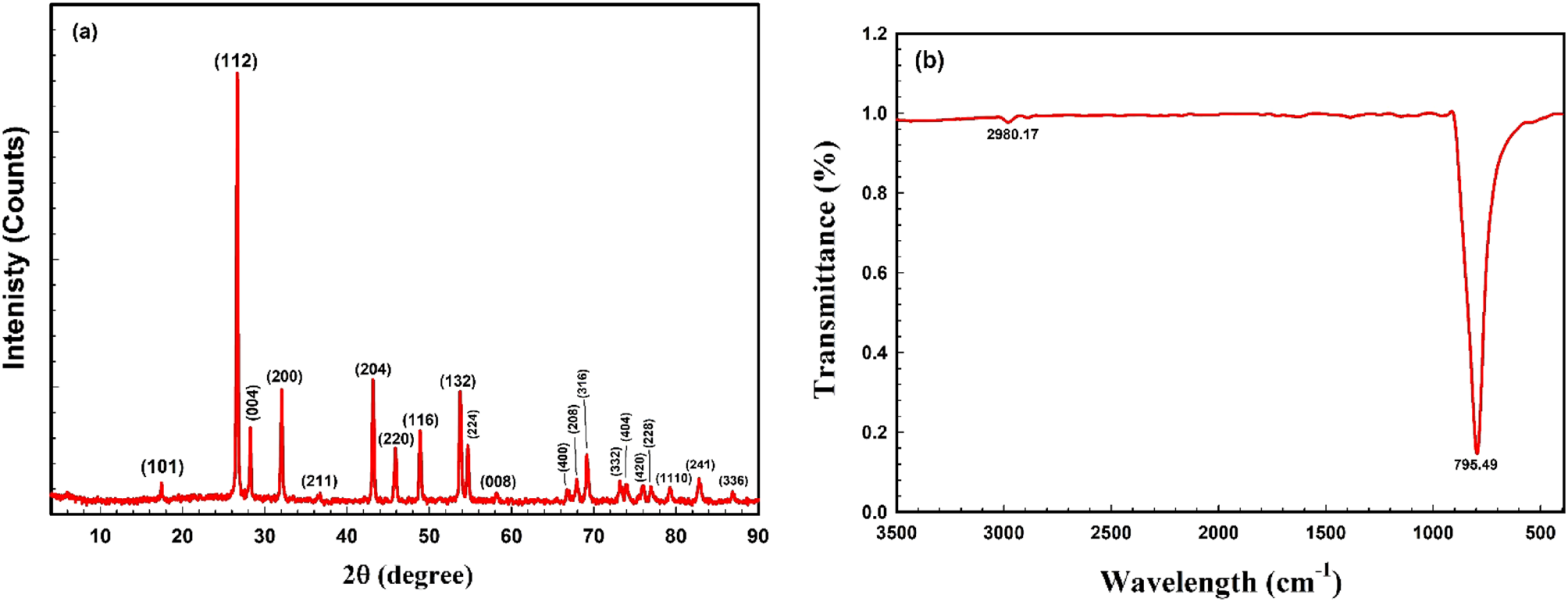
(**a**) XRD pattern and (**b**) FTIR spectrum of BWO nanocrystals.

X-ray diffraction was used to analyze the phase composition and crystal structure of BWO nanostructures. The powder XRD pattern of the synthesized BWO nanostructures is shown in Fig. 2a. The pattern's diffraction peaks correlate to the (101), (112), (004), (200), (211), (204), (220), (116), (132), (224), (008), (400), (208), (316), (332), (404), (420), (228), (1110), (241), planes of BWO, which is demonstrated by JCPDS card number 72–0746. The XRD pattern reveals the synthesis of pure single-phase BWO with a tetragonal phase and is similar to those presented in the literature^28,32,33^.

The FT-IR characterization approach was employed to evaluate the chemical bond formation and numerous interactions between the BWO nanostructures. In Fig. 2b, FT-IR spectra of BWO have been demonstrated. The primary absorption bands include 795 and 2980 cm^−1^. The intense band at 795 cm^−1^ is typical of the ${WO}_{4}^{2-}$ group's W–O vibration. The MO bonds of a solid oxide system are responsible for this band^34^. Since the nanoparticles were synthesized in an aqueous solution, the peak at 2980 cm^-1^ might be attributed to the vibrations of the sample's adsorbed water molecules^35^. These findings match well with those obtained in^29–31^.

**Figure 3:**
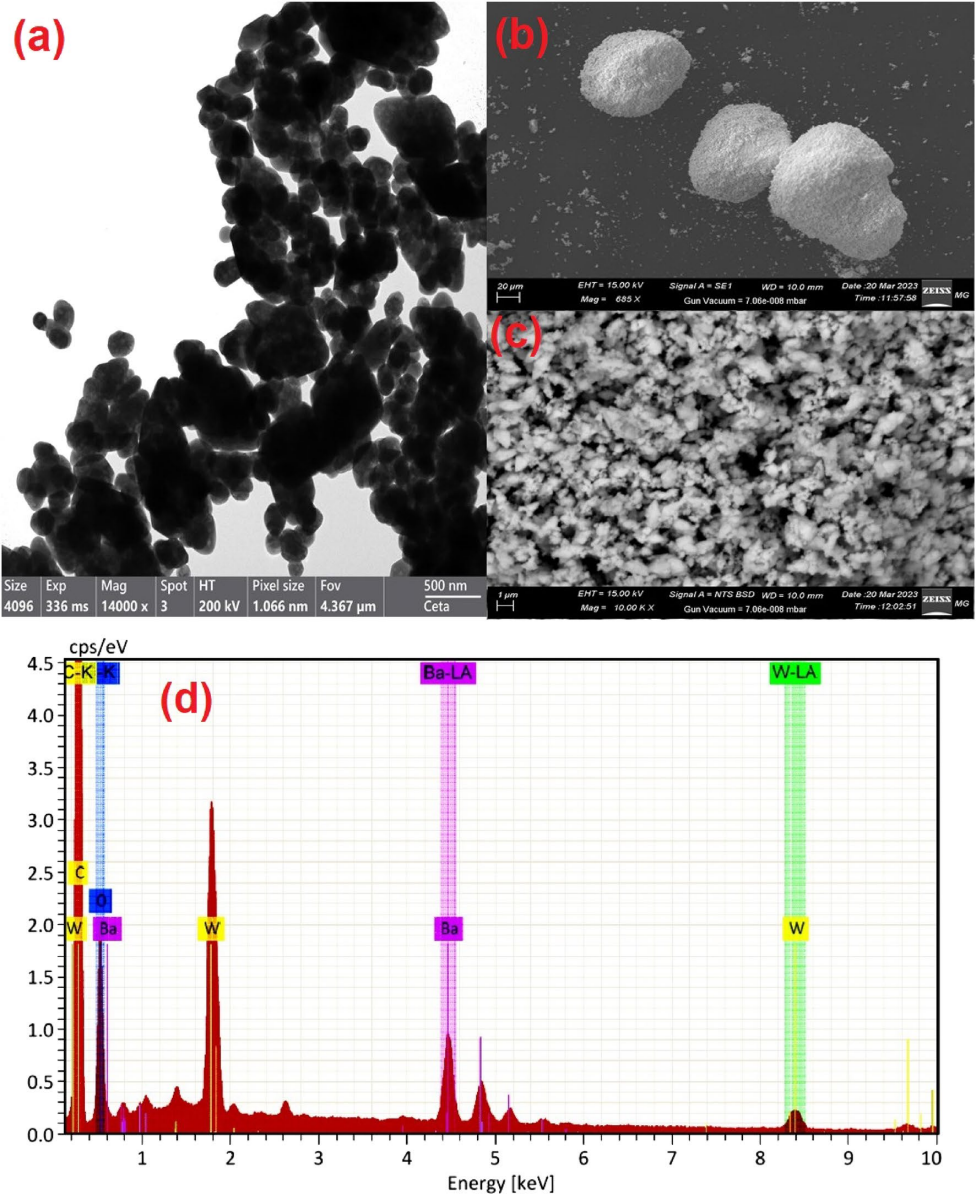
(**a**) TEM image, (**b** & **c**) SEM images, and (**d**) EDX spectra of the BWO nanospheres.

**Figure 4:**
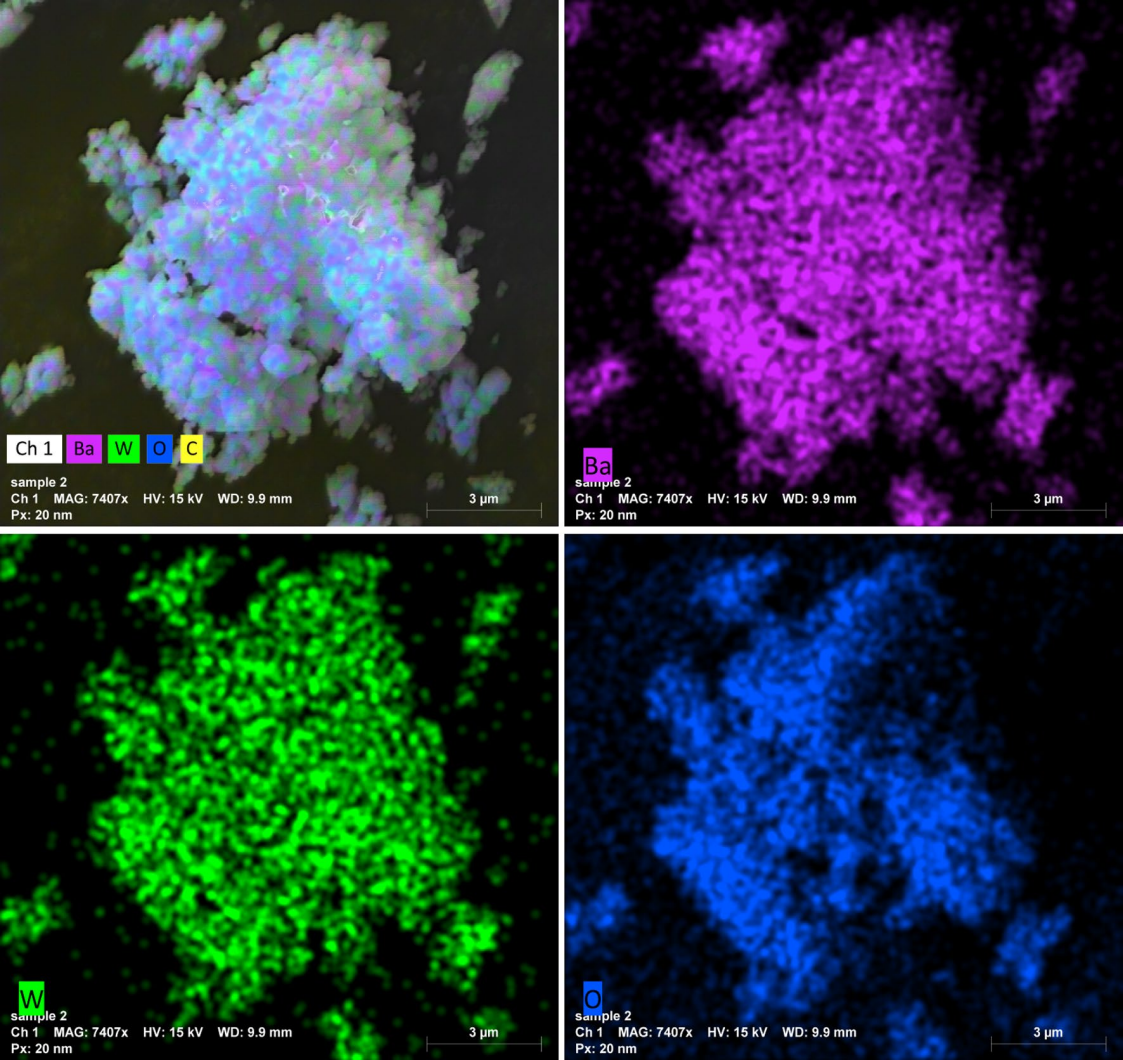
Mapping images of BWO nanocrystals.

TEM and SEM extensively investigated the BWO NPs to identify the actual size and shape of the particles. The TEM image of BWO shows the formation of sphere-shaped structures in the nanoscale range (see Fig. 3a). Figure 3b,c showed characteristic scanning electron microscopy (SEM) micrographs of BWO nanostructures at various magnifications. SEM micrographs revealed the uniform sphere-like structural characteristics of the synthesized BWO. A low-magnification SEM image demonstrates the excessive production of nanospheres. Small aggregations of nanospheres are detected with higher resolution, which might be due to inter-particle contact during the synthesis process. A close examination of nanospheres shows their rough outer surface, which might be attributable to tiny holes formed during development. Figure 3d depicts the EDX spectra of a BWO nanosphere. The figure demonstrated that the Ba, W, C, and O are present in stoichiometric quantities without any foreign elements, demonstrating the purity of the BWO nanocrystals. The appearance of carbon (C) may originate from the environment. Furthermore, the mapping images revealed that all elements are distributed uniformly throughout the BWO nanocrystals, as presented in Fig. 4.

### Determination of point of zero charge

**Figure 5:**
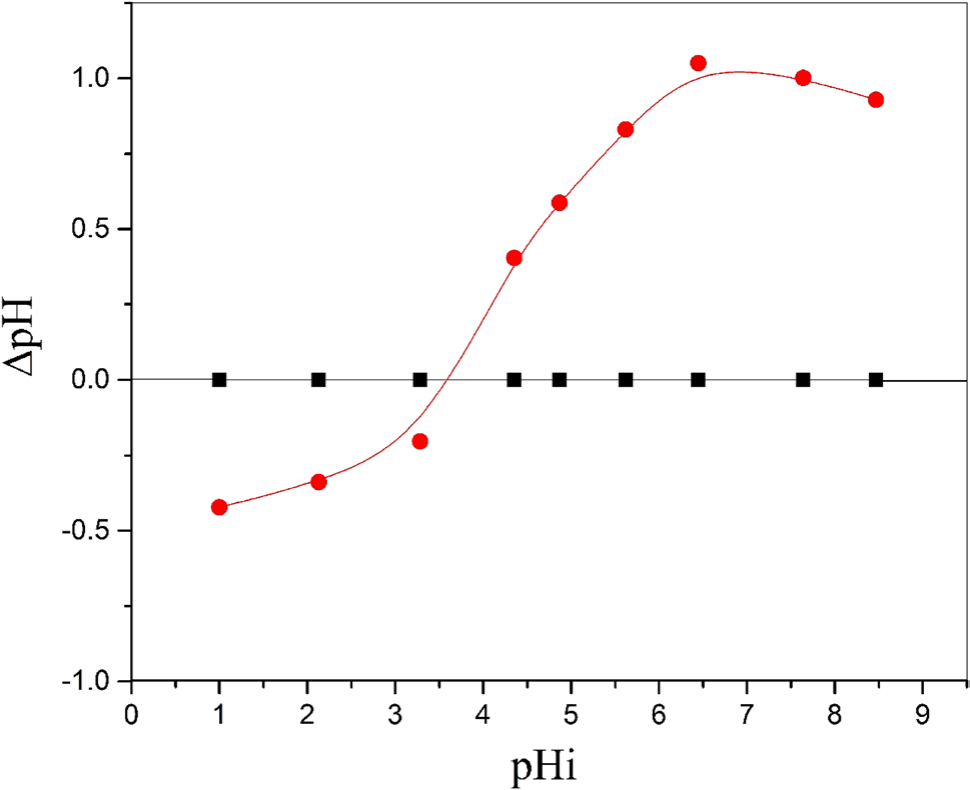
Determination pH_pzc_ for BWO nanocrystals.

The point of zero charges, pHpzc, is defined as the point at which the net charge of the material surface is zero; above it, the surface charge is negative, and below it, the surface charge is positive. The low value of pHpzc indicates that the material is a promising sorbent since it has a wide range of pH values at which the surface has a negative charge. Hence, it can attract the cations in a wide range of pH values. Figure 5 displays a relation between the initial pH, pHi, and pH_F_. The point at which the value of pHi is equal to pH_F_ (ΔpH = 0) was recorded as pHpzc of BWO surface and found to be 3.5. Therefore, at a pH higher than 3.5, the sorption of radionuclides should be high.

### Cobalt and europium adsorption

#### Effect of pH

**Figure 6:**
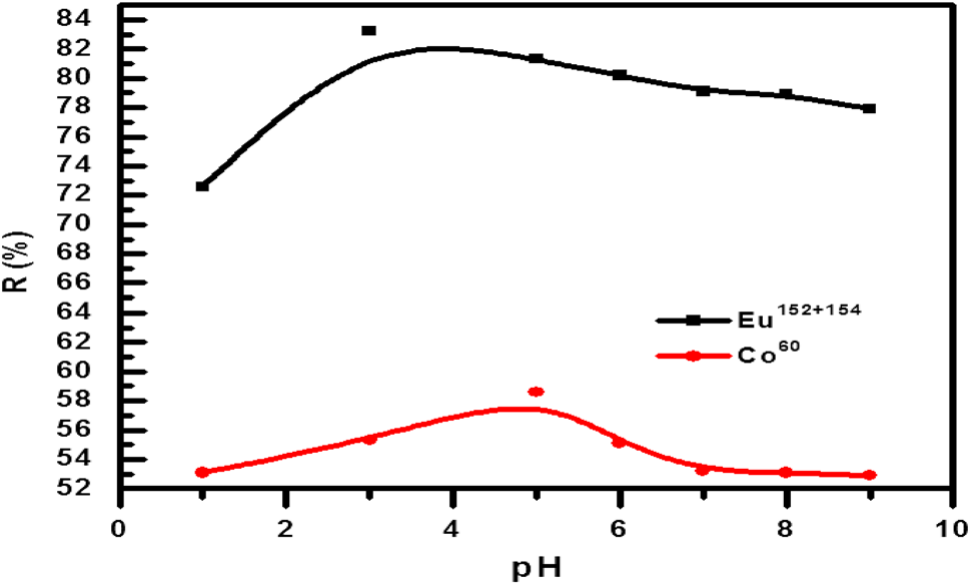
Effect of pH on the ^60^Co and ^152+154^Eu (II) sorption by BWO nanocrystals.

**Figure 7:**
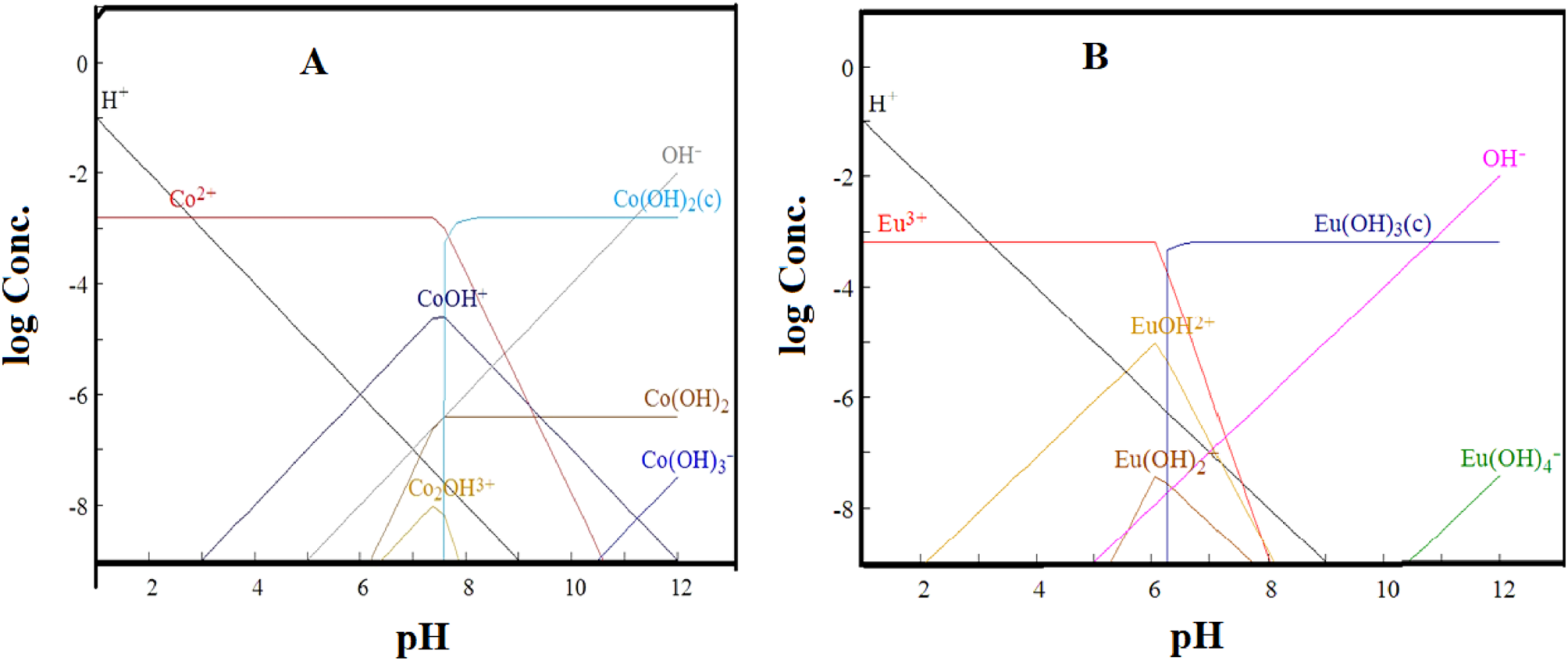
The speciation of (**A**) cobalt and (**B**) europium on the sorption ions at different pH values.

The pH effect on eliminating radioactive cobalt and europium by the BWO has been studied at the pH domain from 1.0 to 9.0, as shown in Fig. 6**.** It is clear that the uptake of both radionuclides is pH dependent, and the removal percentage of the both radionuclide adsorbed onto the BWO increase with increasing the values of pH. At the low pH values, the uptake of a radionuclide has been inhibited; this may be due to the existence of a hydrogen proton (H^+^) that competes with the radioactive cobalt and europium for the adsorption sites onto the BWO surface^36^. The speciation was done using Hydra/Medusa chemical equilibrium software^13^ at 100 mg/L initial metal ions concentration at room temperature and pH range 1–12. From the speciation diagram, Fig. 7A, the divalent species, Co^2+^, is the predominant cobalt species at pH below 7.6; above this value, the cobalt ion is precipitated as Co(OH)2. Therefore, the total cobalt removal at pH < 7.6 is mainly attributed to the sorption process, while the precipitation process is the major mechanism at pH > 7.6. The monovalent species, CoOH^+^, is presented at a pH value greater than 3. This means that the divalent species, Co^2+^, and the monovalent species, CoOH^+^, coexist at a pH range of 3–7.6. While for europium ions, the trivalent species, Eu3 + , is the dominant species till pH 6, it starts to precipitate as Eu(OH)_3_ species at pH above 6 as displayed in Fig. 7B**)**. Whereas Eu(OH)^2+^ and Eu(OH)_2_^+^ species are presented at the pH range of 2.3–8 and 5.3–7.6, respectively. The pH 5.0 for both cations was selected as the optimum pH value for sorption to avoid precipitation of metal ions in the form of their hydroxides.

#### Impact of contact time

**Figure 8:**
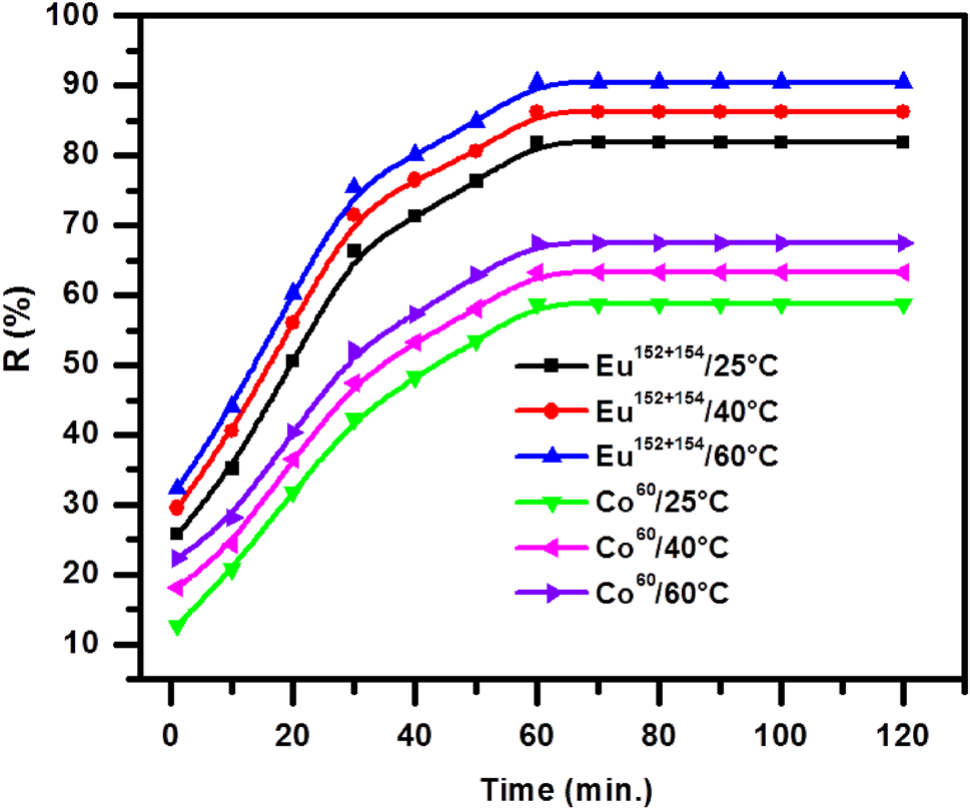
Effect of shaken time on the sorption of ^60^Co and ^152+154^Eu onto the BWO nanocrystals.

The sorption work has been studied at several temperatures, a definite amount from the prepared BWO nanospheres, 0.01 g, and the most favorable pH equal to 5. Acquired data of the radioactive cobalt and europium has been designated in Fig. 8. It is also evident from the figure that the uptake of each of the radioactive cobalt and europium increases with the increase of time, which starts at a high rate and then to approximately reduced. The bending point is after 40 min; the adsorption process progresses slowly until the equilibrium state is reached after 60 min. Then, the adsorption of radioactive elements has been in a steady state. The amount of europium adsorbed on the surface of the BWO nanospheres was greater than that of adsorbed cobalt; this signalizes that the hydrated ions of both cobalt and europium affect the adsorption process. The europium atomic radius is greater than the cobalt atomic radius. So, the hydrated radius for ^152+154^Eu is lower than the hydrated radius for ^60^Co, and this means that the highest atomic radius becomes the littlest hydrated, so the quantity of europium adsorbed onto the BWO nanospheres becomes higher. The effect of temperature on the adsorption process was also studied, and it was found that the higher the temperature, the greater the adsorption of europium and cobalt, which indicates that this process is endothermic.

### Sorption kinetic studies

**Figure 9:**
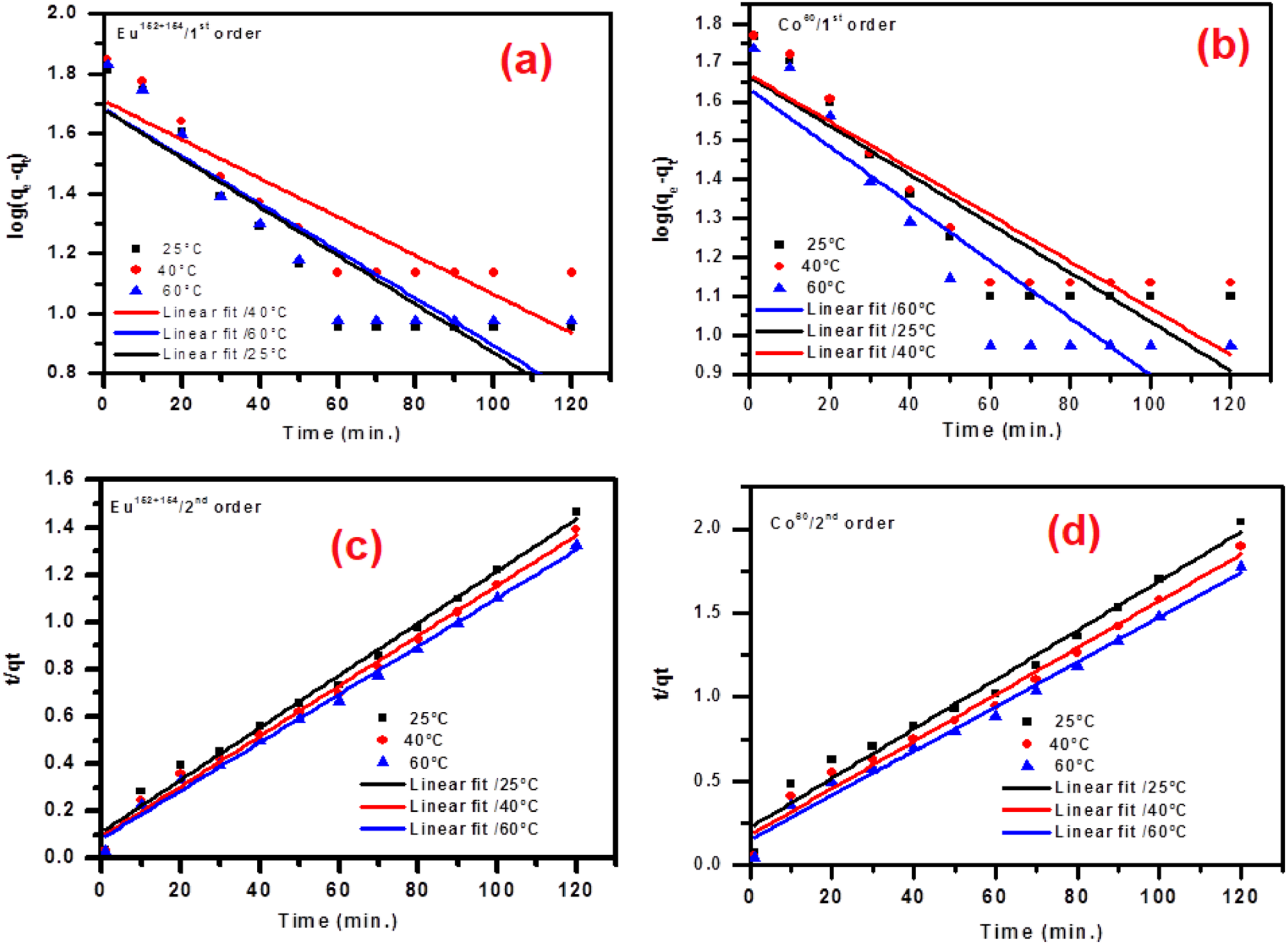
Pseudo-first order (**a**, **b**) and Pseudo-second order (**c**, **d**) plots for the adsorption of ^60^Co and ^152+154^Eu onto the BWO nanocrystals.

Kinetics studies for cobalt and europium radionuclide sorption onto the BWO nanospheres were analyzed utilizing four kinetic models: pseudo-first order, pseudo-second order, and intra-particle diffusion, as well as Elovich models. The Lagergren equation (pseudo-first order expression) has been shown as follows^37^:

3 $$\log \left( {q_{e} - q_{t} } \right) = \log q_{e} - \frac{{k_{1} }}{2.303}t$$

where qe and qt (mg/g) are the amount of the radionuclide sorbed at equilibrium and at time t, respectively, and k_1_ is the rate constant of the pseudo-first-order model (min^-1^). Figure 9a,b shows the linear relationship between log (qe-qt) and t. The intercept and slope were used to determine the calculated sorption capacities at equilibrium (qe) and rate constant (k_1_) of the first-order kinetic model, respectively. The qe and k_1_ values and the correlation coefficients (R^2^) values of each radionuclide are given in Table 1. The linearity relation produced from the figure of the pseudo-first-order kinetic model suggests the applicability of the pseudo-first-order kinetic model to appropriate the experimental results through the initial period of the sorption procedure. Also, the values of calculated sorption capacities at equilibrium, qe, must conform with the values of the experimental data of the sorption process. Table 1 shows that the linear correlation coefficients of the sorption process are excellent; the values calculated qe do not agree with experimental qe for both studied radionuclides. Therefore, it could be suggested that the sorption of both radionuclides onto prepared BWO nanospheres is not a first-order reaction. The pseudo-second-order has been demonstrated in the following^6^:

4 $$\frac{t}{{q_{t} }} = \frac{1}{{k_{2} \;q_{e}^{2} }} + \frac{1}{{q_{e} }}t$$

**Table 1:** Adsorption kinetic parameters for Co(II) and Eu(III) adsorption onto the BWO nanocrystals.

Kinetic models	Parameters	Co^60^		Eu^152+154^			
		25 °C	40 °C	60 °C	25 °C	40 °C	60 °C
	q_e__(exp.)_(mgg-1)	58.8	63.3	67.7	81.9	86.3	90.5
Pseudo-first order equation	q_e__(Cal.)_ (mgg^-1^)	5.28	5.31	5.11	5.38	5.53	5.39
	K_1_(min^-1^)	0.014	0.014	0.016	0.018	0.014	0.016
	R^2^	0.921	0.915	0.921	0.918	0.900	0.914
Pseudo-second order equation	q_e__(Cal.)_ (mgg^-1^)	61.4	66.9	70.9	87.9	90.5	93.4
	K_2_ (min^-1^)	0.001	0.001	0.001	0.001	0.001	0.001
	R^2^	0.979	0.984	0.987	0.990	0.992	0.993
Intra-particle diffusion	K_p_ (mgg^-1^ min^-1/2^)	5.27	5.24	5.26	6.32	6.28	6.39
	C	10.37	15.23	19.28	24.4	29.3	32.44
	R^2^	0.928	0.929	0.937	0.937	0.947	0.955
Elovich model	β (mg g^-1^ min^-1^)	0.087	0.086	0.085	0.073	0.071	0.069
	*α* (g mg^-1^)	18.19	27.91	39.58	48.7	67.1	81.04
	R^2^	0.919	0.917	0.927	0.939	0.905	0.909

The k_2_ refers to the rate constant of the pseudo-second-order, g mg^-1^ min^-1^. The plots of t/qt versus t for each 60Co and 152þ154Eu radionuclides at various temperatures are given in Fig. 9c,d. The high correlation coefficient (R^2^), and the linear relationship between t/qt and t explain that the sorption behavior of each radionuclide follows pseudo-second-order kinetics. The correlation coefficient values (R^2^) (> 0.99) are high, as well as the calculated sorption capacity (qe) at equilibrium is proportionate with the experimental data. These results illustrate that the rate of the pseudo-second-order model is prevalent, and the chemical sorption process controls the sorption behavior for each radionuclide^10^.

Elovich equation has been applied successfully for describing the second-order model by claiming that the effective solid surface is energetically heterogeneous.

5 $$q_{t} = (\frac{1}{\beta })\ln (\alpha \beta ) + (\frac{1}{\beta })\ln t$$

**Figure 10:**
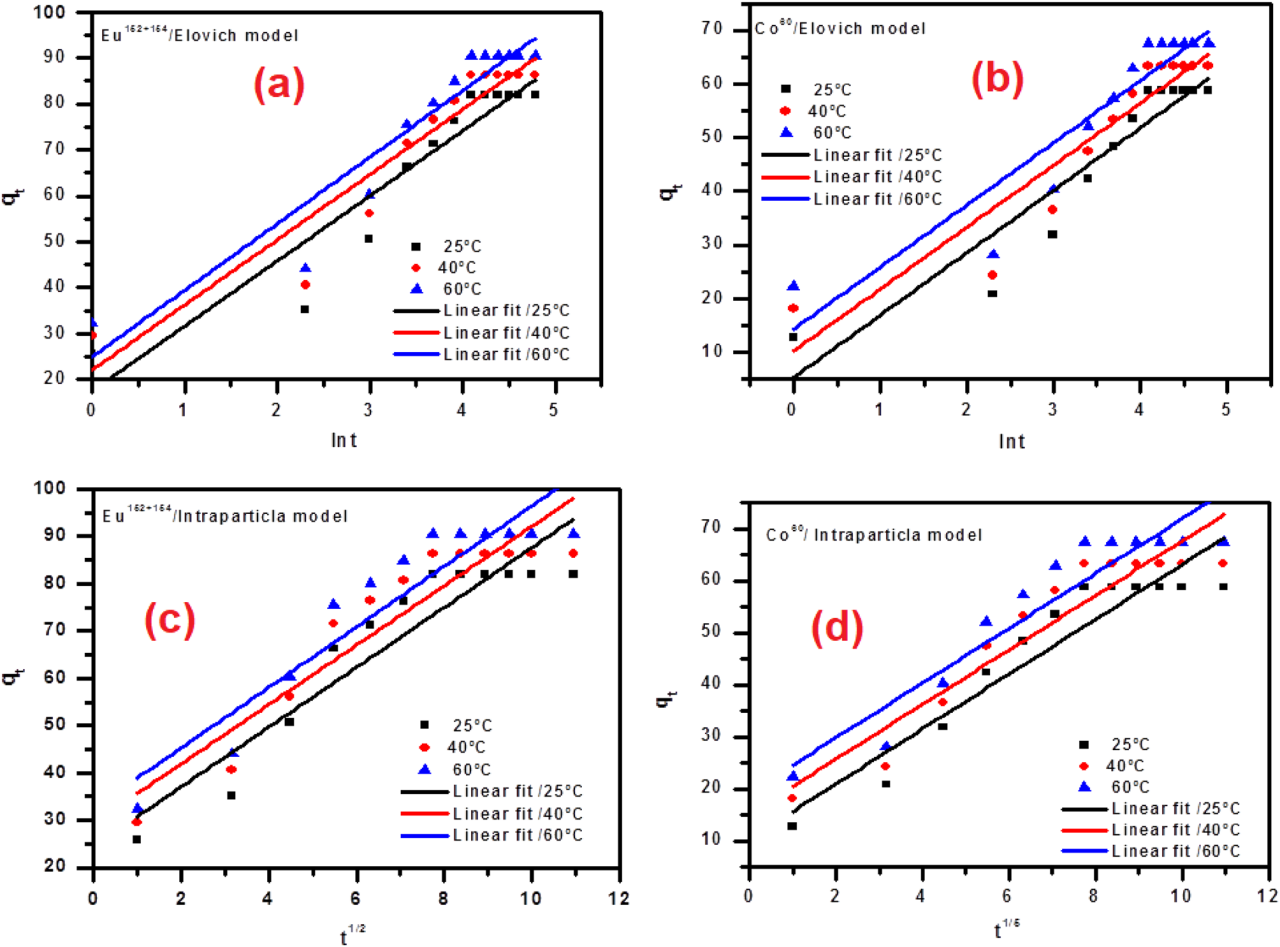
Adsorption kinetics models: (**a** & **b**) Elovich Kinetics and (**c** & **d**) Intraparticle diffusion kinetics plots for the adsorption of ^60^Co and ^152+154^Eu onto the BWO.

The symbols α and β are referred to as constants of the Elovich equation. α (mg g^-1^ min^-1^) points to the rate of adsorption; also, β refers to the desorption constant (g mg^-1^). A relation between q_t_ and ln t to the adsorption of radioactive cobalt and europium has been displayed in Fig. 10a,b. The constants of this have been listed in Table 1. As the adsorption temperature increased, the values of α increased, and the values of β decreased. The value of β is connected with the range of surface coverage. Increasing the initial rate for the adsorption process is based on the temperature that decreases the accessibility of the adsorption surface towards the adsorbate. The obtained data indicates that the adsorption rate could be amplified using increasing temperature. The trend about β is proportionate with the data obtained from conventional systems of activated chemisorption; this points out that β is a signal to several available sites on the surface of the BWO nanospheres for the adsorption process. The intraparticle diffusion model elucidates ions migration from the bulk of aqueous medium towards the surface of sorbent material. This model has been used to display the adsorption process for studied ions onto the BWO. The intraparticle model has been clarified by Weber–Morris utilizing the following expression:

6 $$q_{t} = K_{di} t^{1/2} + C$$

The K_di_ points out the rate constant of this model (mg/g min^1/2^), and C refers to the constant that articulates the thickness of the boundary layer. The intraparticle diffusion rate was obtained from the plots qt versus t^1/2^, as shown in Fig. 10c,d. According to this model, the plotting graphic of qt versus t^1/2^ gives a straight line. It can be assumed that the involved mechanism of the sorption process is controlled by intraparticle diffusion. The values of intercept C give an idea about the boundary layer thickness, i.e., the larger the intercept, the greater the boundary layer effect. Values of C and K_di_, the intraparticle diffusion rate constant, are given in Table 1 for cobalt and europium.

### Isotherms studies

**Figure 11:**
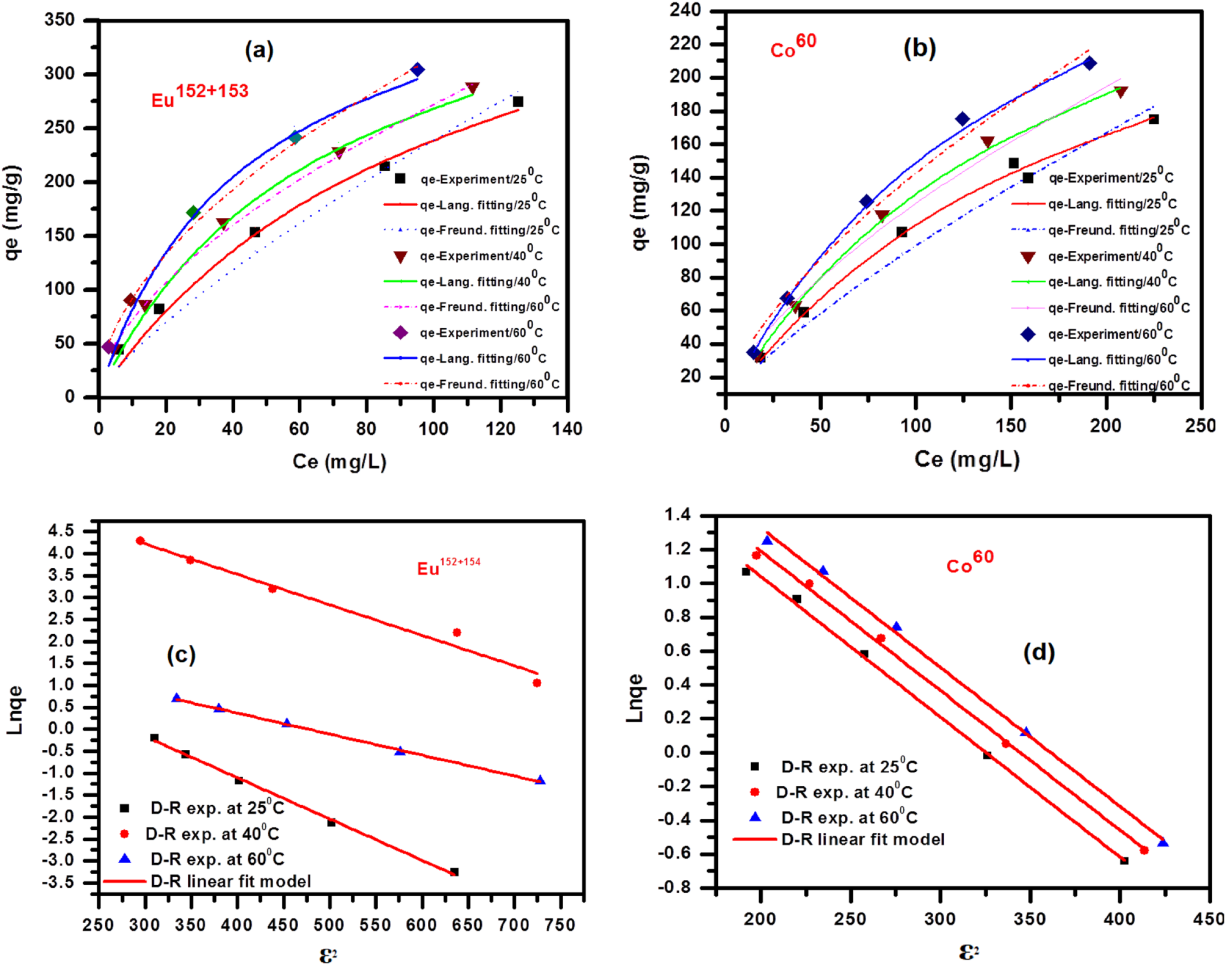
isotherm data (**a** & **b**) Langmuir and Freundlich (**c** & **d**) Dubinin-Radushkviech (D-R) models of ^152+154^Eu and ^60^Co adsorption onto the surface of the BWO at different temperatures.

Equilibrium studies have been commonly reported using several isotherm equations that clarified the surface's characterization and affinity of adsorbent BWO material at a specific temperature and certain pH. The equilibrium models depict the relevance between quantities of the adsorbate on the surface of the adsorbent BWO nanospheres and the concentration at equilibrium for adsorbate^38^. In the present work, the isotherm studies of the radioactive cobalt and europium uptake from waste stream solutions onto the BWO nanospheres at diverse temperatures have been estimated. Figure 11 displays the isotherm data obtained by different models, such as Langmuir and Freundlich, alongside the Dubinin-Radushkviech (D-R) models.

#### Langmuir model

Langmuir model is considered the monolayer coverage for the sorbent surface and also supposes that the uptake process executed onto the homogeneous surface of the adsorbent the BWO nanospheres and each active site have been energetically congruent^7^. The non-linear expression for this model can be written as:

7 $$q_{e} = \frac{{Q^{0} bC_{e} }}{{1 + bC_{e} }}$$

where q_e_ represented the amount of radioactive cobalt and europium sorbed onto the BWO nanospheres (mg/g), C_e_ pointed out the concentration of cobalt and europium at equilibrium, mgL^-1^, and Q^o^ symbolized the monolayer capacity for the adsorption (mg g^-1^) as well as b is referred to the constant of this model relating to the energy of the adsorption (b α e^-ΔG/RT^).

The graphic relationship between q_e_ and C_e_ for both radioactive cobalt and europium sorbed onto the BWO nanospheres, offered in Fig. 11a,b, corroborative that the equation of this model is a sensible depiction of chemisorption isotherm. Obtained data for constants Q^o^ and b that are estimated have been shown in Table 2.

**Table 2:** Different isotherm models including Langmuir, Freundlich and D-R models affection adsorption of ^152+154^Eu and ^60^Co onto the surface of the BWO at different temperature.

Metal ions	Temp °C	Q° (mg/g)	Langmuir parameters			*1/n*	Freundlich parameters		q_m_ (Mol/Kg) _mol/g_	D-R		
			b (L/mg)	R_L_	R^2^		k_*f*_	R^2^		β mol^2^ /kJ^2^	E kJ^/^mol	R^2^
^**152+15**^**Eu**	25	409.9	0.011	0.468	0.988	1.34	7.64	0.982	9.77	0.009	9.45	0.997
	40	425.2	0.017	0.363	0.990	1.78	20.9	0.999	10.12	0.005	10.13	0.977
	60	454.3	0.027	0.268	0.987	1.95	29.8	0.999	14.21	0.004	12.2	0.998
^**60**^**Co**	25	310.6	0.006	0.632	0.999	1.35	3.25	0.969	14.8	0.008	8.91	0.998
	40	336.3	0.007	0.604	0.999	1.59	6.96	0.988	17.1	0.008	9.91	0.997
	60	363.7	0.007	0.581	0.999	1.61	7.99	0.986	19.6	0.008	11.91	0.997

The constants of Langmuir model Q^o^ and b were determined for each radionuclide and the values are shown in Table 2. It is clear that from Table 2, the monolayer sorption capacity (Q^o^) values of BWO nanospheres towards ^152+154^Eu radionuclide are relatively higher than that of ^60^Co radionuclide. The constants of the Langmuir model Q^o^ and b for the sorption of each radionuclide increased with temperature, indicating that the capacity of sorption process and intensity of sorption are enhanced as temperatures increased. As the temperature increases the monolayer sorption capacity increases; this suggests that the active surface available for sorption has been increased with temperature. Also, the Langmuir isotherm model can be used to determine the dimensionless parameter at equilibrium or the separation factor, R_L_ (R_L_ = 1/1 + bC_o_), where, Co is the initial concentration of radionuclides (mg/L). The computed value of RL shows the type of isotherm to be irreversible (R_L_ = 0), favorable (0 < R_L_ < 1), linear (R_L_ = 1), or unfavorable (R_L_ > 1). Table 2 shows the values of R_L,_ which are greater than 0 and less than 1, indicating the favorable sorption isotherms of each radionuclide.

#### Freundlich model

Freundlich isotherm is extracted to model the multilayer sorption and adsorption onto heterogeneous surfaces. The non-linear equation of this model can be displayed as follows^15^:

8 $$q_{e} = k_{f} C_{e}^{1/n}$$

K_f_ (mg/g), a constant, refers to the relative capacity for sorption of the BWO nanospheres and 1/n points out intensity of this process. The graphical illustration between q_e_ and C_e_ has been offered in Fig. 11a,b. Table 2 displays the obtained values of the Freundlich parameters (n and K_f_). It is clear that from the figure the sorption of each radionuclide obeys the Freundlich model through the entire range of concentration studied. The Freundlich isotherm constants K_f_ and 1/n are determined; the results are shown in Table 2. The values of 1/n assess the intensity of sorption and the heterogeneous nature of the surface. The nearer the value to zero, the more the anticipated heterogeneity of the sorbent surface; 1/n greater than one entails chemical cooperative sorption ^12,15^.

#### Dubinin–Radushkviech isotherm (D–R isotherm)

The nature of the sorption processes was studied using the D-R isotherm model^15^:

9 $$\ln \;q_{e} = \ln \;q_{m} - \beta \varepsilon^{2}$$

where q_m_ symbolizes the maximum amount from radionuclides sorbed on unit mass from nano barium tungstate surface (sorption capacity, mmol/g), while β points out the constant concerning the energy of sorption (mol^2^/kJ^2^), as well as is $\varepsilon$ Polanyi potential [$\varepsilon$ = RT ln (1 + 1/Ce)], gas constant, R, (kJ/mol K), as well as T is the temperature in kelvin (K).

Mean free energy (E) values for the sorption process can be determined from the following expression:

10 $$E={(-2\beta )}^{-1/2}$$

Information about the sorption mechanism, whether this mechanism is chemical or physical, is determined by the values of E. If the magnitude of E is ranged from 8 to 16 kJ/ mol, the sorption process is controlled by a chemical mechanism^15^. At the same time, the physical mechanism affects sorption if the magnitude of E is less than 8.0 kJ/mol. The plots of lnqe versus ε^2^ for the sorption of both radionuclides at various temperatures are shown in Fig. 11c,d. The linear relation elucidates that the D-R isotherm successfully described the investigated sorption results. The D-R parameters are evaluated and shown in Table 2. The D-R parameters are evaluated and shown in Table 2. The mean free energy values (E) of sorption process is in all temperature used in the range of 8–16 kJ/mol, which are within the energy ranges of a chemisorption process^13^.

#### Effect of temperature

The evaluation of thermodynamic parameters is fundamental to comprehension of the nature as well as the spontaneity of the sorption technique^38^. In this work, the thermodynamic parameters can be calculated by using the Vant Hoff equation to the obtained data at various temperatures as follows:

11 $${\text{ln K}}_{{\text{c}}} = \, \frac{{\Delta {\text{S}}^{^\circ } }}{{\text{R}}} - \frac{{\Delta H^{^\circ } }}{RT}$$

Kc represents the equilibrium sorption constant, R refers to the gas constant, and T points out the temperature in kelvin (K). Kc, for all applied temperatures, has been computed using the product of Langmuir equation parameters (Q° and b)^6^.

**Figure 12:**
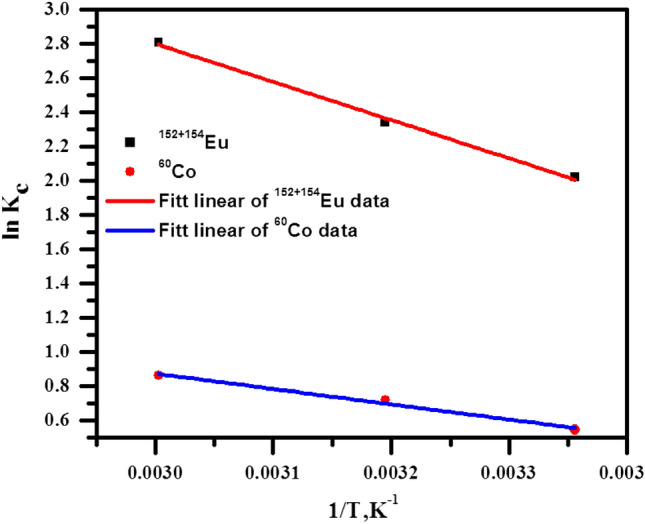
Plotting relation between lnK_c_ with 1/T of ^152+154^Eu and ^60^Co adsorption onto the surface of the BWO.

Figure 12 shows the plotting relation between lnK_c_ with 1/T supply with a straight line, ΔS°, and ΔH^o^ have been computed from the intercept and slope of this line. It is clear that from Table 3, the endothermic nature of the sorption process can be indicated by the positive sign of ΔH^o^ values. While values of ΔS° has a positive sign, this is because of the exchange of radionuclide with more mobile species present onto the BWO nanospheres; this means increasing the entropy during the sorption of radionuclides^39–41^. Also, to investigate the spontaneity of this sorption process, another thermodynamic parameter was calculated, Gibbs free energy, from the relationship between ΔH° and ΔS° as illustrated below:

12 $$\Delta G^{^\circ } = \Delta H^{^\circ } - T\Delta S^{^\circ }$$

**Table 3:** Thermodynamic parameters for the adsorption of ^152+154^Eu and ^60^Co onto the surface of the BWO nanocrystals.

Radioactive metal	∆G, kJ/mol			∆S, J/mol.K	∆H, kJ/mol
	298 K	313 K	333 K		
^152+154^Eu	− 11.44	− 14.60	− 18.82	210.87	51.40
^60^Co	− 3.34	− 4.55	− 6.15	80.18	20.55

The obtained values of the ΔG° are listed in Table 3. It was clear that the sorption process of ^60^Co and ^152+154^Eu is spontaneous because the values of ΔG° had a negative sign at all temperatures.

#### Regeneration of BaWO_4_

The loaded BaWO_4_ is used for repeated utilization after regeneration via the desorption of metal ions. After the sorption process, the saturated sorbent by radioactive cobalt and/or europium was separated by filtration to remove the traces of the mother liquor and dried to constant weight. A certain 0.1 g of the dried BaWO_4_ after cobalt and/or europium sorption was shaken with 10 mL of nitric acid solution with different molarities (0.1, 0.3, and 0.5 mol/L). Sorption and desorption processes were repeated several times, and the activity of each radionuclide was detected in each case. This method was repeated using NaOH of the same molarities as used for HNO_3_. The desorption percent, %D, can be computed using the following equation:

13 $$D\% = \frac{{C_{a} \times 100}}{{C_{s} }}$$

where C_a_ and C_s_ refer to concentrations of the both radionuclides in an aqueous solution and solid phase, respectively.

It was established that the regenerated BaWO_4_ can be used for several cycles (Five cycles) until completely exhausted; the results are reported in Table 4. The desorption percent in the first cycle is 93.1 and 96.6% for ^152+154^Eu and ^60^Co, respectively, using 0.3 mol/L HNO_3,_ which was desired as the best eluent. The removal percent was also computed in each cycle to determine the efficiency of BaWO_4_; it decreased in each cycle, as reported in Table 4. The experimental outcomes suggested that BaWO_4_ is a potential sorbent for removing cobalt and europium radionuclides with repeated usage.

**Table 4:** Regeneration of ^60^Co and ^152+154^Eu using 0.3 mol/L of nitric acid and/or NaOH.

Cycle no.	Sorption, %		Desorption, %			
	^152+154^Eu	^60^Co	HNO_3_, 0.3 mol/L		NaOH, 0.3 mol/L	
			^152+154^Eu	^60^Co	^152+154^Eu	^60^Co
Cycle 1	92.3	84.1	93.1	96.6	72.1	76.2
Cycle 2	90.5	82.4	84.4	92.5	64.2	69.3
Cycle 3	83.1	72.1	72.5	75.1	53.6	65.3
Cycle 4	60.3	55.3	55.5	63.9	47.1	57.6
Cycle 5	44.3	39.6	49.6	51.5	40.5	45.6

### Comparison of the sorption capacity with different sorbents

The comparison of sorption capacities of ^152+154^Eu and ^60^Co radionuclides by variable materials gained from the available literature is reported in Table 5. The sorption capacity alteration is due to each sorbent's properties, such as surface area, particle size, porosity, and functional groups. The value of sorption capacity achieved in this work is greater than that of several other sorbents. This revealed that BaWO_4_ is considered an encouraging sorbent for removing ^60^Co and ^152+154^Eu radionuclides from radioactive waste.

**Table 5:** Comparison of the sorption capacity for ^152+154^Eu and ^60^Co radionuclides using various sorbents.

Sorbents	Capacity, mg/g		References
	^152+154^Eu	^60^Co	
BWO nanocrystals	409.9	310.6	Present work
C@ZrO_2_/MnMgZnFe_2_O_4_	136.98	82.51	^39^
Titanium oxide incorporated in polyacrylonitrile	136.3	70.5	^42^
Al^3+^ and Fe^3+^ doped zirconium and titanium phosphates	20	NR*	^43^
Magnetic graphene oxide	NR	147	^44^
Resin on the base of di-(tert-butylbenzo)-18-crown-6	NR	9.8	^45^
diethylenetriaminepentaacetic acid/silica dioxide	192.4	NR	^46^
polyaniline/zirconium aluminate	175.7	140.3	^47^

*NR refers to not reported.

## Conclusion

Synthetic barium tungstate BWO nanocrystals have been examined as inorganic sorbents for the uptake of ^60^Co and ^152+154^Eu from the waste stream. The kinetics studies of each radionuclide have been studied, and acquired data investigated utilizing different kinetic models. Obtained data elucidated that a pseudo-second-order mechanism is prevailing. The sorption has been controlled by a chemical mechanism. Different isotherm models estimated equilibrium isotherms, and the results were successfully modeled utilizing Langmuir and Freundlich, as well as Dubinin-Radushkviech (D-R) models. According to the D-R model, the maximum sorption capacity and the mean free energy of the studied ions have been determined. The sorption of both radionuclides is an endothermic process and spontaneous in nature. These obtained data showed that synthetic barium tungstate nanocrystals are promising sorbent materials for removing ^60^Co and ^152+154^Eu from the waste stream.

## Data Availability

All data generated or analyzed during this study are included in this published article.
